# Associations Between Inflammatory and Bone Turnover Markers and Mortality in Hemodialysis Patients

**DOI:** 10.3390/biomedicines13051163

**Published:** 2025-05-10

**Authors:** Alexandru Florin Sircuța, Iulia Dana Grosu, Adalbert Schiller, Ligia Petrica, Viviana Ivan, Oana Schiller, Felix-Mihai Maralescu, Marcel Palamar, Monica-Nicoleta Mircea, Daniel Nișulescu, Ionuț Goleț, Flaviu Bob

**Affiliations:** 1 Department of Internal Medicine II—Nephrology University Clinic, “Victor Babes” University of Medicine and Pharmacy, Eftimie Murgu Sq. No. 2, 300041 Timișoara, Romania; alexandru.sircuta@umft.ro (A.F.S.); schiller.adalbert@umft.ro (A.S.); petrica.ligia@umft.ro (L.P.); mihai.maralescu@umft.ro (F.-M.M.); marcel.palamar@umft.ro (M.P.); bob.flaviu@umft.ro (F.B.); 2 Centre for Molecular Research in Nephrology and Vascular Disease, Faculty of Medicine, “Victor Babes” University of Medicine and Pharmacy, Eftimie Murgu Sq. No. 2, 300041 Timișoara, Romania; 3 County Emergency Hospital, L. Rebreanu Street, Nr. 156, 300723 Timișoara, Romania; ivan.viviana@umft.ro; 4 Department of Internal Medicine II—Cardiology University Clinic, “Victor Babes” University of Medicine and Pharmacy, Eftimie Murgu Sq. No. 2, 300041 Timișoara, Romania; 5 B Braun Avitum Dialysis Centre, 300417 Timișoara, Romania; oana.schiller@bbraun.com; 6 Institute of Cardiovascular Diseases Timișoara, 13A Gheorghe Adam Street, 300310 Timișoara, Romania; mircea.monica@yahoo.ro (M.-N.M.); daniel.nisulescu@umft.ro (D.N.); 7 Research Center of the Institute of Cardiovascular Diseases Timisoara, 13A Gheorghe Adam Street, 300310 Timișoara, Romania; 8 Department of Management, Faculty of Economics and Business Administration, University of the West, 300115 Timișoara, Romania; ionut.golet@e-uvt.ro

**Keywords:** CKD-MBD, inflammation, hemodialysis, mortality, parathyroid hormone, soluble Klotho

## Abstract

**Background/Objectives:** Chronic kidney disease–mineral and bone disorder (CKD-MBD) and systemic inflammation contribute to mortality in hemodialysis (HD) patients. The primary aim of this study was to determine whether specific CKD-MBD markers and inflammatory biomarkers are associated with increased mortality risk in HD patients. **Methods:** We conducted a retrospective cohort study on 63 stage 5D CKD patients undergoing maintenance HD. Serum intact parathyroid hormone (iPTH), soluble Klotho, calcium, phosphorus, 25(OH)D (25-hydroxyvitamin D), transforming growth factor-beta (TGF-β), vascular endothelial growth factor (VEGF), C-reactive protein (CRP), and interleukin-6 (IL-6) were analyzed. A Cox regression analysis assessed mortality predictors, and linear regression analysis evaluated CKD-MBD–inflammation correlations. **Results:** Lower iPTH (<329.3 pg/mL) levels were the only significant mortality predictor (*p* = 0.042). Other CKD-MBD markers (calcium, phosphorus, 25(OH)D, VEGF, TGF-β) did not impact survival. Soluble Klotho correlated positively with IL-6 (r = 0.57, *p* < 0.001), suggesting a compensatory inflammatory response. **Conclusions:** Our findings demonstrate that low iPTH levels and advanced age are independent predictors of mortality in hemodialysis patients. The positive association between soluble Klotho and IL-6 suggests a potential compensatory inflammatory response. These results highlight the need for further research to clarify underlying mechanisms and to explore novel therapeutic strategies.

## 1. Introduction

Chronic kidney disease (CKD) is a progressive condition affecting over 10% of the global population. Its prevalence is rising due to risk factors like obesity, hypertension, and diabetes. As CKD advances, morbidity and mortality increase, reducing life expectancy and quality of life, especially for those with the need for dialysis. Early detection and effective risk management are crucial to slowing progression and improving patient outcomes [1,2]. Extracorporeal dialysis is the most widely used renal replacement treatment for patients with end-stage renal disease (ESRD) [3]. Currently, an estimated 3.9 million patients are enrolled in dialysis programs worldwide, with 89% undergoing hemodialysis [2,4,5].

CKD often leads to mineral bone disorder (CKD-MBD), affecting osteogenesis, bone metabolism, and vascular calcifications. This complication starts in the early stages and worsens with renal replacement therapy, making it a significant concern in CKD management [6].

CKD-MBD involves changes in calcium, phosphorus, parathormone, and vitamin D levels. Low levels of vitamin D_3_ increase Fibroblast growth factor-23 (FGF-23), leading to hyperparathyroidism and bone remodeling. Elevated FGF-23 is associated with worsening kidney function and higher mortality, regardless of bone remodeling [7,8,9,10,11].

Soluble Klotho protein (sKlotho) regulates FGF-23 and calcium–phosphorus metabolism. A deficiency in this protein is linked to premature aging, vascular calcification, cardiac hypertrophy, and mineral bone disease (MBD) in both mice and humans [12,13].

Chronic inflammation in hemodialysis patients increases morbidity and mortality, driven by elevated cytokines like transforming growth factor beta (TGF-β) and vascular endothelial growth factor (VEGF). TGF-β regulates renal fibrosis and CKD progression, while VEGF enhances endothelial permeability, promotes adhesion molecule expression, and links immune inflammation to angiogenesis [14,15,16].

In addition to this, VEGF is associated with endothelial dysfunction, left ventricular hypertrophy, and endothelial dysfunction and with a higher risk of mortality, which is also associated with serum inflammation markers, although its involvement in hemodialysis (HD) patients is less known [17,18,19,20]. There are few studies in the context of CKD-MBD, and the data on bone density show a directly proportional association between VEGF and bone density, although some studies show a lack of association between the two elements [21,22].

There is a cumulative risk associated with the traditional risk factors for CKD, including hypertension and diabetes, as well as factors related to CKD-MBD, such as serum levels of the Klotho protein, serum intact parathyroid hormone (iPTH), and vitamin D. Additionally, inflammatory factors like VEGF and TGF-β also influence mortality.

The primary objective of this study was to evaluate the associations between baseline levels of CKD-MBD markers (including iPTH, calcium, phosphorus, 25(OH)D, and soluble Klotho) and inflammatory biomarkers (IL-6, CRP, TGF-β, VEGF) with overall mortality in hemodialysis patients. We specifically aimed to determine whether iPTH and soluble Klotho serve as independent predictors of survival outcomes.

## 2. Materials and Methods

We conducted a retrospective cohort study to assess the impact of inflammatory markers and CKD-MBD markers on mortality, as well as the association between these two types of markers in patients undergoing hemodialysis.

### 2.1. Patient Population

The study group consisted of 63 CKD stage 5D patients, all of whom were receiving maintenance hemodialysis at the Avitum B. Braun Centre in Timişoara, Romania. These patients had been receiving chronic HD for over one year but less than five years, with a mean dialysis vintage of 3.3 ± 1 years. Inclusion in the study required signed informed consent. Patients were excluded if they had experienced an infection within the previous six months, had heart failure with reduced ejection fraction (HFrEF), had undergone a change in their renal replacement technique or dialysis facility, or were scheduled for kidney transplantation within the next three months.

The Ethics Committee of Victor Babes University of Medicine and Pharmacy, Timisoara, Romania, approved all procedures, which were conducted in compliance with the Declaration of Helsinki and the institutional research committee’s ethical standards (protocol code 33/30.06.2021). The Ethics Committee of the Dialysis Center approved this study. Eligible patients were asked to give written informed consent before any study procedures were carried out.

### 2.2. Clinical and Biochemical Assessment

In this study, fasting blood samples were obtained immediately before the mid-week hemodialysis session, using either an arteriovenous fistula or a central venous catheter. The samples were maintained at 4 °C for approximately one hour prior to centrifugation at 1000× *g* for 10 min. Post-centrifugation, serum aliquots were prepared and stored at −80 °C until further analysis.

The analysis included serum creatinine, blood urea, and inflammatory markers such as plasma TGF-β, VEGF, and IL-6 and anemia parameters including complete blood count, ferritin, and transferrin saturation (TSAT) and markers of CKD-MBD (sKlotho, iPTH, serum calcium, serum phosphorus). Serum levels of sKlotho were measured using a sandwich ELISA technique, employing the Abbexa™ Anti-Klotho Antibody kit. This kit is based on a sandwich enzyme-linked immunosorbent assay, utilizing a horseradish peroxidase (HRP)-conjugated anti-Klotho antibody as the detection antibody. The reaction was visualized using tetramethylbenzidine (TMB) substrate. The protocol followed the manufacturer’s instructions for the quantitative determination of soluble Klotho levels in patient serum samples, while the Enzyme Immunoassay method measured the plasma levels of TGF-β and VEGF.

Dialysis adequacy was evaluated by equilibrated Kt/V (eKt/V), which was calculated based on pre- and post-dialysis urea concentrations using standard formulas, accounting for post-dialysis urea rebound.

For each patient, we collected demographic information (including gender and age), medical history, dialysis duration, etiology of CKD, and a history of comorbidities such as diabetes mellitus.

After the dialysis session, patients’ weights were measured, and this dry weight value was used to calculate their body mass index (BMI).

All data collected during the baseline visit were included in an interim analysis, which served as a retrospective evaluation of the participants in this study.

### 2.3. Follow-Up

For all HD patients, we tracked overall mortality as our key endpoint, measuring outcomes 48 months after inclusion.

### 2.4. Statistical Analysis

Continuous variables were summarized as mean ± standard deviation (SD) if normally distributed or as median if non-normally distributed. Categorical variables are reported as count (percentage). The normality of the continuous data was assessed using the Kolmogorov–Smirnov test in MedCalc v12.5.0 (Mariakerke, Belgium). Between-group comparisons of categorical data employed Pearson’s chi-square test, and ordinal associations were evaluated with Spearman’s rank correlation. For normally distributed continuous variables, Scheffé’s test was used for all pairwise comparisons and Levene’s test to verify the equality of variances.

To identify optimal survival cut-offs for each continuous biomarker, we used the maximally selected rank statistic in Jamovi v2.3 (Jamovi Project; https://www.jamovi.org, accessed on 29 April 2025). After determining each cut-point, Kaplan–Meier median, 1-, 3-, and 5-year survival estimates were generated.

All statistical computations and graphics were performed in R v4.1, 2021 (R Core Team, Vienna, Austria). Group differences in survival were assessed using log-rank, Gehan, Tarone–Ware, and Peto–Peto tests, and hazard ratios with 95% confidence intervals were obtained from Cox proportional hazards models.

## 3. Results

### 3.1. Characteristics of the Study Group

The characteristics of the patient groups among the 63 HD patients included in our study are presented in Table 1.

### 3.2. Survival Analysis

To evaluate if inflammatory markers and CKD-MBD markers are associated with mortality in HD patients, we conducted a Cox regression analysis using a stepwise backward selection method. Variables with *p* < 0.10 in the group comparison were included in the final model.

To evaluate the association between inflammatory markers, CKD-MBD markers, and mortality in hemodialysis (HD) patients, we conducted a Cox regression analysis using a stepwise backward selection method. Variables with a significance level of *p* < 0.05 in the group comparisons were retained in the final model. We ensured that the assumptions of the Cox model were checked during our analysis (Table 2).

In the univariate Cox analysis, an iPTH < 329.3 pg/mL at referral was the only statistically significant variable identified as a risk factor for mortality (*p* = 0.045). The other variables did not show any tendency toward significance. In the multivariate Cox analysis, iPTH remained the only significant factor for mortality (*p* = 0.042) (Table 2).

Using the Cox proportional hazards regression analysis to identify predictors of mortality, we found that certain cut-off values were associated with a significantly lower probability of survival. Once the cut-off values were established, we calculated the median survival as well as the 1-, 3-, and 5-year survival rates.

#### 3.2.1. IPTH Impact on Survival

**Figure 1:**
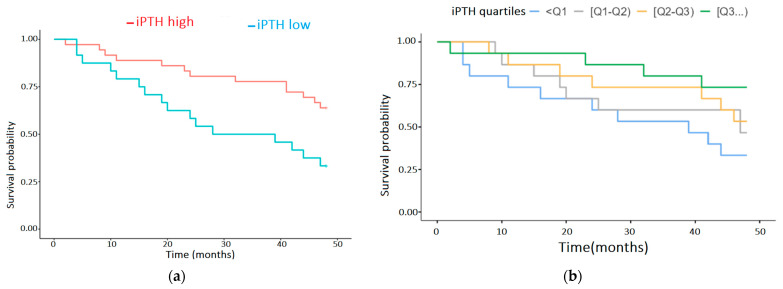
(**a**) Cox regression analysis of the influence of serum IPTH on survival of HD patients; (**b**) Cox regression analysis of the quartiles of serum IPTH.

We performed survival analyses on serum iPTH, which emerged as a significant predictor in our Cox regression (Figure 1). The optimal iPTH cut-off of 329.3 pg/mL was determined automatically in Jamovi v2.3 using the maximally selected rank statistic, which evaluates every possible division of the continuous variable and selects the threshold that maximizes the log-rank statistic (with adjustment for multiple testing). In our cohort, this resulted in a value of 329.3 pg/mL. In Figure 1a, the survival rate is significantly lower at 3 (50 vs. 76%, log-rank *p* < 0.001) and 4 years (27% vs. 64%, log-rank test *p* < 0.001) in patients with an iPTH value < 329.3 pg/mL. In Figure 1b, the extreme quartile groups, Q1 (iPTH < 273.9 pg/mL) and Q4 (iPTH > 719.8 pg/mL), emphasize the disparity in survival probabilities. Hence, Q1 shows the poorest survival, and Q4 shows the most favorable outcomes. Although the overall log-rank test between these two groups did not reach significance (*p* = 0.15), both the Tarone–Ware and Peto–Peto tests were highly significant (*p* < 0.001), supporting the hypothesis that very low iPTH levels may be associated with reduced survival.

To focus on the poorest outcomes, we combined all of Q1 and the lower half of Q2 (iPTH < 329.3 pg/mL), yielding a low-iPTH group of 23 patients. Only 8 (34.8%) had survived at 4 years, while 15 (65.2%) had passed away. On the other hand, in the high-iPTH group (≥329.3 pg/mL), the remaining 37 patients from Q2 to Q4 included 23 survivors (62.2%) and 14 deaths (37.8%) at 4 years. As a result, the lowest half of Q2, together with Q1, captures the subset of patients with the worst long-term outcomes (iPTH < 329.3 pg/mL).

#### 3.2.2. Impact of 25(OH)D, 25-Hydroxyvitamin D(Calcidiol) on Survival

To ascertain the median survival rates and the 1-, 3-, and 5-year survival rates, we established a cut-off point for serum 25(OH)D based on a survival outcome of 47.4 IU/L. This cut-off point did not indicate a significantly low survival rate (*p* = 0.743).

When 25(OH)D levels were elevated, the 12-month survival rate was 57% [30–100.0%, 95% CI]. In contrast, the 36-month survival rate for this group reached its lowest point at 29% [9–92.2%, 95% CI].

Conversely, when 25(OH)D levels were low, the 12-month survival rate increased to 89% [81–97.7%, 95% CI], while the 36-month survival rate experienced a slight decline to 72% [61–85.2%, 95% CI].

#### 3.2.3. Impact of Serum Phosphorus (PO_4_) on Survival

To illustrate the impact of serum PO_4_ on survival, a cut-off point of 3.87 was calculated, even if a notably decreased survival rate was not found (*p* = 0.901). All the same, when serum PO_4_ was high, the 12-month survival rate reached a peak of 90% [82–98.71%, 95% CI], in contrast to the 36-month survival, which dropped gradually to 74% [63–87.21%, 95% CI]. In comparison, the 12-month survival rate declined significantly to 64% [41–99.47%, 95% CI], while the 36-month survival rate reached its minimum at 36% [17–79.47%, 95% CI] for decreased PO_4_ levels.

#### 3.2.4. Impact of Serum Calcium on Survival

For serum calcium, a cut-off point of 8.48 mg/dL was established to assess its influence on survival time, but it showed no significant impact on survival (*p* = 0.526). Nevertheless, the analysis indicated that when serum calcium levels were high, the 12-month survival rate was 80.6% [68.6–94.58%, 95% CI], while the 36-month survival rate gradually decreased to 61.1% [47.1–79.30%, 95% CI]. In contrast, for low serum calcium levels, the 12-month survival rate increased slightly to 92.0% [82.0–100.00%, 95% CI], and the 36-month survival rate rose gradually to 76.0% [61.0–94.73%, 95% CI].

#### 3.2.5. Impact6 of Plasma TGF-β on Survival

In spite of the impact on mortality not being notable (*p* = 0.422) for plasma TGF-β, it was split into two groups after determining a cut-off point of 22.2 pg/mL to calculate the median survival rates and 1-, 3-, and 5-year survival rates. High values revealed better survival rates, not only for the 12-month survival rate, which was increased to a maximum of 88.89% [77.8–100.0%, 95% CI], but also the 36-month survival rate, which decreased minimally to 77.78% [63.6–95.2%, 95% CI]. Nevertheless, for low levels, the 12-month survival rate was 78.57% [59.8–100.0%, 95% CI], making it similar to the 36-month survival rate, which reached the lowest point at 57.14% [36.3–89.9%, 95% CI].

#### 3.2.6. Impact of Plasma VEGF on Survival

Despite the impact on mortality not being meaningful (*p* = 0.802), the cut-off for plasma VEGF was determined to be 1041 pg/mL, and the median survival along with the 1-, 3-, and 5-year survival rates were calculated. By dividing plasma VEGF levels into two groups—those with increased values and those with decreased values—we assessed the impact on survival. For increased values, the 12-month survival rate was 74.1% [59–92.6%, 95% CI]; conversely, the 36-month survival rate was 55.6% [40–77.8%, 95% CI], which remained unchanged. For decreased values, the 12-month survival peaked at 100.0% [100–100.0%, 95% CI], while the 36-month survival also remained stable at 94.1% [84–100.0%, 95% CI].

#### 3.2.7. Impact of Soluble Klotho on Survival

The impact of soluble Klotho on survival was assessed after establishing a cut-off point of 482. Median survival rates, along with the 1-, 3-, and 5-year survival rates, were calculated by categorizing patients into high and low soluble Klotho groups. In the high Klotho group, the 12-month survival peaked at 100.00% [100.0–100.0%, 95% CI], while the 36-month survival slightly decreased to 83.33% [58.3–100.0%, 95% CI]. For the low Klotho group, the 12-month survival was 83.78% [72.7–96.5%, 95% CI], and the 36-month survival dropped to 67.57% [54.0–84.5%, 95% CI]. Nevertheless, the overall impact on survival was not significant (*p* = 0.254).

#### 3.2.8. Impact of Age on Survival

A survival analysis using age as a continuous variable demonstrated that higher age was significantly associated with an increased mortality risk. In the univariate Cox regression analysis, age was a significant predictor of mortality (HR = 1.07; 95% CI: 1.01–1.12; *p* = 0.016). The multivariate Cox regression analysis confirmed age as an independent predictor of mortality (HR = 1.12; 95% CI: 1.01–1.23; *p* = 0.024), indicating that each additional year of age was associated with a 7–12% increase in mortality risk.

### 3.3. Association Between Inflammation and CKD-MBD

To determine the link between CKD-MBD markers and inflammation markers, linear regression was employed as it serves as a robust statistical method for analyzing relationships between continuous variables. These correlations suggest potential predictive relationships that linear regression can quantify. By modeling these links, linear regression not only helps in understanding the strength and direction of the associations but also allows confounding variables to be controlled, enhancing the reliability of the findings. This method is particularly suited for this analysis, as it helps identify and quantify the impact of CKD-MBD markers on inflammation markers, thereby elucidating potential underlying mechanisms in chronic kidney disease.

The analysis revealed significant associations, notably between soluble Klotho baseline and plasma IL-6 baseline, and between serum PO_4_ and serum iPTH baseline. The specific inflammatory and CKD-MBD markers included in the model are presented in Table 3.

Further, we employed the Pearson’s chi-squared test to compare categorical variables and Spearman’s rank correlation test to assess the relationship between ordinal variables. Plasma IL-6 levels at baseline showed a strong statistically significant correlation with soluble Klotho (r = 0.57, *p* = 0.001), while soluble Klotho showed a positive correlation with hemodialysis efficiency (eKt/V) (r = 0.26, *p* = 0.04).

### 3.4. Association Between iPTH and Age

A Pearson correlation analysis was performed to assess the relationship between patient age and baseline serum intact parathyroid hormone (iPTH) levels.

The results showed no statistically significant correlation (r = −0.229, *p* = 0.078), suggesting that age and iPTH contribute independently to patient survival outcomes.

## 4. Discussion

The relationship between iPTH levels and patient survival in individuals undergoing HD has garnered increasing interest in the nephrology literature.

The findings from our study show a significant relationship between baseline iPTH levels and survival in HD patients. Specifically, higher iPTH levels correlate with better survival outcomes over 36 months. Patients with low iPTH levels (below 329.3 pg/mL) experienced significantly reduced survival, especially at 36 months, where survival dropped to 50%, compared to 77.8% in those with higher iPTH levels. Additionally, it should be noted that, in general, high levels of iPTH are considered a negative prognostic factor.

Comparing our study’s findings on plasma iPTH levels and mortality with those of Aquino et al. [23], key points emerge for discussion. Our research identified a critical threshold of 329.3 pg/mL for iPTH, below which survival rates decreased significantly. Patients with high iPTH levels had better survival rates compared to those with low levels, suggesting an increased risk of early mortality at lower levels. Aquino et al. emphasize monitoring iPTH levels but focus mainly on incident dialysis patients. This difference in study populations is crucial, as mineral metabolism dynamics can vary significantly due to the initiation of dialysis, potentially affecting the prognostic significance of iPTH [23].

Both studies highlight the importance of monitoring iPTH for managing mineral metabolism in end-stage renal disease. However, our findings suggest that low iPTH levels represent a clinically relevant risk factor, challenging traditional views on iPTH levels.

These results align with another investigation, which emphasizes the prognostic significance of iPTH levels in clinical practice. The investigation underscores the fact that abnormal iPTH levels can serve as indicators of underlying health issues, particularly in patients with chronic conditions such as kidney disease [24]. Both our study and this investigation highlight the detrimental effects associated with low iPTH levels, reinforcing the idea that maintaining optimal hormone levels is crucial for improving patient survival.

Moreover, the statistical significance of our findings (*p* = 0.042) further supports the assertion made in the article that iPTH levels are not merely biomarkers but are actively involved in the pathophysiology of mortality risk. The progressive decline in survival rates with lower iPTH levels, as illustrated in our results, complements the article’s discussion on the multifaceted role of parathyroid hormone in regulating various physiological processes, including bone metabolism and cardiovascular health [24].

Lower baseline iPTH levels (<329.3 pg/mL) were associated with increased mortality, consistent with the “adynamic bone disease” paradigm, where suppressed parathyroid activity correlates with vascular calcification and poor outcomes [4,25]. Our Kaplan–Meier assessment illustrated that those with an iPTH level over the 329.3 pg/mL threshold had a definite survival advantage. This result, which is in line with previous investigations [5,26], emphasizes how crucial it is to prevent excessive iPTH suppression when managing CKD-MBD.

In our cohort, lower baseline iPTH levels were associated with reduced 4-year survival, with survival improving progressively with higher iPTH values. These findings differ somewhat from the DOPPS Practice Monitor results [27], where extremely high PTH levels (>600 pg/mL) were associated with increased mortality (HR 1.17, *p* = 0.007). In the DOPPS results, the PTH levels rose substantially after 2010, with a significant proportion of patients, particularly Black patients, exceeding 600 pg/mL.

Although DOPPS identified a threshold above which survival decreased, our data showed an ongoing rise in survival with higher iPTH rather than a U-shaped relationship. These discrepancies could be due to differences in the cohort size, PTH dispersion, therapeutic management, and the patient population [6].

In our study, survival analysis showed that patients with suppressed iPTH levels (<150 pg/mL) had worse outcomes, consistent with findings from the COSMOS study, where lower iPTH levels were linked to increased mortality [28]. In contrast to suppressed iPTH, higher levels of iPTH (>600–700 pg/mL) were related to improved survival in our cohort. This outcome could illustrate the intricate relationship between mineral metabolism and patient outcomes.

Phosphate control substantially changes the influence of iPTH on survival, according to the COSMOS study, proving that even with optimal biochemical parameters, elevated iPTH is not a reliable indicator of mortality [28]. Additionally, the MBD-5D research demonstrated that targeted interventions such as Cinacalcet therapy can improve clinical outcomes, even among patients with severe secondary hyperparathyroidism [29]. In brief, our findings corroborate the existing literature, reinforcing the notion that iPTH levels are a vital determinant of mortality risk. Future research should focus on elucidating the mechanisms by which iPTH influences survival and exploring potential therapeutic interventions aimed at optimizing iPTH levels in at-risk populations.

The strong positive correlation between sKlotho and IL-6 illustrated by the linear regression analysis is intriguing. Klotho is known for its role as an anti-aging protein with anti-inflammatory properties, particularly through suppression of the NF-kB pathway and regulation of oxidative stress responses [1]. While elevated IL-6 typically signifies systemic inflammation, Klotho’s association with higher IL-6 in this cohort may reflect compensatory upregulation in response to inflammatory stress [2].

Klotho is widely recognized for its protective roles against aging, vascular calcification, and oxidative stress. Mechanistically, Klotho suppresses inflammatory responses primarily through inhibition of the nuclear factor-kappa B (NF-κB) pathway, which is a central regulator of pro-inflammatory cytokines, including IL-6 [30]. Klotho deficiency has been shown to upregulate NF-κB activity, leading to increased IL-6 expression and immune cell activation [31]. However, recent studies highlight the context-dependent nature of Klotho’s role in inflammation. For example, a study on HD patients demonstrated positive correlations between Klotho and certain inflammatory cytokines, including IL-12p70 and IL-10, though not IL-6, suggesting selective immune modulation depending on the patient’s inflammatory profile [32]. An investigation into glioma patients reported a positive correlation between serum soluble α-Klotho (sαKlotho) levels and IL-6 (*p* = 0.0347), along with other biomarkers such as VEGF, fractalkine, IFN-γ, GDNF, IL-4, and IL-13. The above findings point to a laborious interaction between inflammatory processes and Klotho, with higher levels of Klotho in glioma patients potentially correlating with higher levels of IL-6 and an expanded inflammatory cytokine profile [33]. In a previous study we revealed that elevated IL-6 is a negative prognostic factor for survival in this patient population [34].

The decline in survival rates for the low Klotho group, particularly at the 36-month mark, emphasizes the need for ongoing monitoring and potential therapeutic interventions aimed at improving Klotho levels in this population to enhance long-term survival outcomes.

The disparity in survival rates observed between the high and low sKlotho groups raises important questions about clinical management in HD patients. The 36-month survival rate of only 67.57% for patients with low Klotho levels suggests that these individuals may be at increased risk for adverse events, potentially due to underlying pathological processes such as vascular calcification and inflammation, which are often exacerbated in CKD [2,35]. High sKlotho levels may offer protective benefits, suggesting its role both as a biomarker and a potential therapeutic target. Enhancing sKlotho levels could improve clinical outcomes and reduce mortality in vulnerable patients, warranting further research.

Contrary to expectations, lower 25(OH)D levels (cut-off: 47.4 IU/L) were linked to better short-term survival (89% at 12 months vs. 57% for higher levels). This paradox may reflect confounding by malnutrition–inflammation complex syndrome (MICS), where inflammation lowers the levels of vitamin D-binding protein and total serum vitamin D [6,36]. The cohort exhibited serum albumin levels within the normal range (mean ± SD: 4.4 ± 0.3 g/dL) and low CRP levels (mean ± SD: 1.0 ± 0.9 mg/dL), indicating minimal systemic inflammation. Additionally, nutritional status appeared to be adequate, as evidenced by stable serum albumin and TSAT levels. These findings suggest that malnutrition and active inflammation, key components of MICS, were unlikely to significantly confound the observed association between iPTH and mortality in this study.

Regarding VEGF, our analysis did not reveal a statistically significant connection between plasma VEGF levels and survival. In agreement with Takayuki’s inquiry, lower VEGF levels were linked to a better prognosis, but raised levels had no discernible effect on survival outcomes. The above results affirm that VEGF has little predictive value for HD patients and point out the need for deeper research into alternative biomarkers [37].

Our results revealed no statistically significant overall association between plasma VEGF levels and mortality in hemodialysis patients. However, the stratified analysis showed that lower VEGF levels were associated with significantly improved survival. This finding aligns with the mechanistic insights of Catar et al. [38], who demonstrated that elevated VEGF reflects underlying inflammation and endothelial dysfunction induced by uremic toxins. Hence, even though VEGF by itself is unlikely to be an accurate predictor of death, its increased levels may indirectly point out a higher inflammatory burden, which is known to be an indicator of poor prognosis in this population. 

Similarly, this investigation revealed a non-significant association between plasma TGF-β and overall mortality (*p* = 0.422) in hemodialysis patients. However, the stratified analysis revealed that higher TGF-β levels correlated with significantly better survival rates. This contrasts with Stefoni et al. [39] who linked lower TGF-β1 levels to increased cardiovascular events. This discrepancy may be due to differing endpoints (mortality vs. cardiovascular events), study populations, and methodological differences in TGF-β measurement.

Our outcomes provide important insights into the differences between Sági et al.’s findings [40] on mortality and inflammatory markers. Ref. [40], who investigated the relationship between inflammation, vascular stiffness (cfPWV), and cardiovascular outcomes. While they observed elevated pro-inflammatory markers (hsCRP, TNF-α, TGF-β1) and depressed anti-inflammatory factors (fetuin-A, α-Klotho, vitamin D3) in their cohort, and found that vascular stiffness correlated with several clinical but not inflammatory markers [40], our results reveal a contrasting association between TGF-β levels and mortality.

According to our observations, preserving iPTH within an ideal range might be essential for enhancing HD patients’ long-term survival. Furthermore, soluble Klotho levels could offer important information about a patient’s inflammatory profile and be a possible biomarker for identifying high-risk individuals. These results may be useful for further research on more individualized treatment strategies, but interventional research is needed to validate these hypotheses. When interpreting our outcomes, it is important to take into account the various limitations of our study. First, our discoveries are not as broadly applicable as they could be due to the single-center setting. In addition, the analysis relied on a baseline measurement of bone and inflammatory markers, which does not account for the fluctuations in these markers throughout time. Third, even after controlling for a number of possible confounders, the association among the markers and mortality could possibly have been altered by additional unmeasured factors. Fourth, the ability of statistical methods to identify subtle effects is limited by the comparatively small sample size.

## 5. Conclusions

In conclusion, lower baseline levels of iPTH and older age were independent predictors of reduced long-term survival in HD patients. Additionally, a positive association between soluble Klotho and IL-6 was observed, suggesting complex interactions between mineral bone disease and systemic inflammation. These findings emphasize the importance of monitoring iPTH levels as part of mortality risk stratification and suggest that soluble Klotho could emerge as a potential biomarker for inflammation-related risk in this population.

## Figures and Tables

**Table 1:** Characteristics of the studied patients.

Parameter	Patients Treated with HD (Mean Values +/− Standard Deviation)
Age (years)	60.1 +/− 11.8
Female: Male	21:42
Hemoglobin (g/dL)	10.9 +/− 1.0
Hematocrit (%)	33.3 +/− 4.3
Serum ferritin (ng/mL)	995.9 +/− 358.0
TSAT (%)	30.6 +/− 10.5
Serum creatinine (predialysis) mg/dL	8.4 +/− 1.9
Serum urea (predialysis) mg/dL	123.1 +/− 28.7
Serum calcium (mg/dL)	8.6 +/− 0.4
	4
Serum intact parathyroid hormone (IPTH) baseline (pg/mL)	551.4 +/− 425.5
Serum phosphorus (PO_4_) (mg/dL)	5.0 +/− 1.3
Plasma TGF-β (pg/mL)	298.9 +/− 609.5
Plasma VEGF baseline (pg/mL)	1371.3 +/− 787.5
Serum albumin (g/dL)	4.4 +/− 0.3
Serum 25(OH)D baseline (ng/mL)	36.7 +/− 10.4
IL-6 (pg/mL)	10.8 +/− 0.9
CRP (mg/dL)	1.0 +/− 0.9
sKlotho (pg/mL)	447.8 +/− 327.2
Serum alkaline phosphatase (ALP) baseline (IU/L)	114.4 +/− 63.7

Legend: HD, hemodialysis; TSAT, transferrin saturation; CRP, C-reactive protein; IL-6, interleukin 6; 25(OH)D, 25-hydroxyvitamin D; iPTH, serum intact parathyroid hormone; TGF-β, transforming growth factor beta; VEGF, vascular endothelial growth factor; sKlotho; soluble Klotho; ALP, serum alkaline phosphatase. The results are expressed as mean values ± standard deviations.

**Table 2:** Univariate and multivariate Cox regression analysis of predictors associated with mortality in HD patients.

	Cox Univariate Regression			Cox Multivariate Regression		
Variables	HR	95% CI	*p*-Value	HR	95% CI	*p*-Value
iPTH	1.02	1.01–1.04	0.045	1.03	1.02–1.05	0.042
Age	1.07	1.02–1.12	0.016	1.12	1.01–1.23	0.024
PO_4_	0.98	0.74–1.31	0.901			
Serum calcium						
soluble KLOTHO	1.01	0.99–1.02	0.254	-	-	-
Serum 25(OH)D	1.01	0.97–1.05	0.743	-	-	-
Plasma TGF-β	1.00	0.98–1.05	0.422	-	-	-
Plasma VEGF	1.00	0.90–1.10	0.802	-	-	-

Legend: iPTH, serum intact parathyroid hormone; TGF-β, transforming growth factor beta 1; VEGF, vascular endothelial growth factor; PO_4_, serum phosphorus; 25(OH)D, 25-hydroxyvitamin D.

**Table 3:** Correlations between CKD-MBD and inflammation markers in linear regression analysis.

Parameter	IL-6 Baseline		CRP Baseline		TGF-β Baseline	
	t	*p* Value	t	*p* Value	t	*p* Value
Baseline soluble Klotho	4.90476	<0.001	−0.35570	0.723	1.99230	0.055
Calcium	0.80389	0.425	−0.31564	0.754	−0.03676	0.971
PO_4_	0.82892	0.411	−0.13309	0.895	−0.57314	0.570
Baseline iPTH	−1.31392	0.195	0.14649	0.884	−1.04538	0.303
Baseline 25(OH)D	−0.01717	0.986	1.73724	0.088	0.85369	0.399

Legend: CRP, C-reactive protein; IL-6, interleukin-6; PO_4_, serum phosphorus; transforming growth factor-β; iPTH, serum intact parathyroid hormone 25(OH)D = 25-hydroxyvitamin D; CKD-MBD, chronic kidney disease–mineral and bone disorder; significance: *p* < 0.05, *p* < 0.01, *p* < 0.001.

## Data Availability

The data presented in this study are available on request from the corresponding author.
